# DEtection of ProxImal Coronary stenosis in the work-up for Transcatheter aortic valve implantation using CTA (from the DEPICT CTA collaboration)

**DOI:** 10.1007/s00330-021-08095-2

**Published:** 2021-06-16

**Authors:** Thomas P. W. van den Boogert, Bimmer E. P. M. Claessen, Maksymilian P. Opolski, Won-Keun Kim, Ashraf Hamdan, Daniele Andreini, Francesca Pugliese, Helge Möllmann, Ronak Delewi, Jan Baan, M. Marije Vis, Adrienne van Randen, Joost van Schuppen, Jaap Stoker, José P. Henriques, R. Nils Planken

**Affiliations:** 1 Department of Clinical and Experimental Cardiology, Amsterdam Cardiovascular Sciences, Amsterdam UMC, University of Amsterdam, Amsterdam, The Netherlands; 2 Department of Cardiology, Noordwest Ziekenhuisgroep, Alkmaar, The Netherlands; 3 Department of Interventional Cardiology and Angiology, National Institute of Cardiology, Warsaw, Poland; 4 Department of Cardiology, Rabin Medical Center, Sackler Faculty of Medicine, Tel-Aviv University, Tel-Aviv, Israel; 5 Department of Cardiology and Radiology, Centro Cardiologico Monzino IRCCS, University of Milan, Milan, Italy; 6 NIHR Cardiovascular Biomedical Research Unit at Barts, William Harvey Research Institute, Barts and The London School of Medicine and Dentistry, Queen Mary University of London & Department of Cardiology, Barts Health NHS Trust, London, UK; 7 Department of Cardiology, St. Johannes-Hospital Dortmund, Dortmund, Germany; 8 Department of Radiology and Nuclear Medicine, Amsterdam Gastroenterology Endocrinology Metabolism, Amsterdam UMC, University of Amsterdam, Amsterdam, The Netherlands; 9 Department of Radiology and Nuclear Medicine, Amsterdam Cardiovascular Sciences, Amsterdam UMC, University of Amsterdam, Meibergdreef 9, 1105 AZ Amsterdam, The Netherlands

**Keywords:** Coronary artery disease, Aortic valve stenosis, Computed tomography angiography, Diagnostic accuracy, Transcatheter aortic valve replacement

## Abstract

**Objectives:**

Computed tomography angiography (CTA) is performed routinely in the work-up for transcatheter aortic valve implantation (TAVI), and could potentially replace invasive coronary angiography (ICA) to rule out left main (LM) and proximal coronary stenosis. The objectives were to assess the diagnostic yield and accuracy of pre-TAVI CTA to detect LM and proximal coronary stenosis of ≥ 50% and ≥ 70% diameter stenosis (DS).

**Methods:**

The DEPICT CTA database consists of individual patient data from four studies with a retrospective design that analyzed the diagnostic accuracy of pre-TAVI CTA to detect coronary stenosis, as compared with ICA. Pooled data were used to assess diagnostic accuracy to detect coronary stenosis in the left main and the three proximal coronary segments on a per-patient and a per-segment level. We included 1060 patients (mean age: 81.5 years, 42.7% male).

**Results:**

On ICA, the prevalence of proximal stenosis was 29.0% (≥ 50% DS) and 15.7% (≥ 70% DS). Pre-TAVI CTA ruled out ≥ 50% DS in 51.6% of patients with a sensitivity of 96.4%, specificity of 71.2%, PPV of 57.7%, and NPV of 98.0%. For ≥ 70% DS, pre-TAVI CTA ruled out stenosis in 70.0% of patients with a sensitivity of 96.7%, specificity of 87.5%, PPV of 66.9%, and NPV of 99.0%.

**Conclusion:**

CTA provides high diagnostic accuracy to rule out LM and proximal coronary stenosis in patients undergoing work-up for TAVI. Clinical application of CTA as a gatekeeper for ICA would reduce the need for ICA in 52% or 70% of patients, using a threshold of ≥ 50% or ≥ 70% DS, respectively.

**Key Points:**

• *Clinical application of CTA as a gatekeeper for ICA would reduce the need for ICA in 52% or 70% of TAVI patients, using a threshold of ≥ 50% or ≥ 70% diameter stenosis*.

• *The diagnostic accuracy of CTA to exclude proximal coronary stenosis in these patients is high, with a sensitivity of 96.4% and NPV of 98.0% for a threshold of ≥ 50%, and a sensitivity of 96.7% and NPV of 99.0% for a threshold of ≥ 70% diameter stenosis*.

• *Atrial fibrillation and heart rate did not significantly affect sensitivity and NPV. However, a heart rate of < 70 b/min during CTA was associated with a significantly improved specificity and PPV*.

**Supplementary Information:**

The online version contains supplementary material available at 10.1007/s00330-021-08095-2.

## Introduction

Coronary artery disease (CAD) is a common concurrent condition in patients with aortic valve stenosis undergoing transcatheter aortic valve implantation (TAVI). Pre-procedural screening for CAD is recommended by the current TAVI guidelines and is usually performed with invasive coronary angiography (ICA) [1–3]. However, pre-procedural revascularization with percutaneous coronary intervention (PCI) is only recommended to consider in patients with coronary stenosis of more than 70% diameter stenosis (DS) in proximal coronary artery segments [3]. Consequently, the majority of patients undergo ICA solely for the exclusion of severe proximal coronary stenosis. This invasive test is associated with a risk of complications and high consumption of healthcare resources. An alternative non-invasive diagnostic test to rule out obstructive CAD is computed tomography angiography (CTA), which is routinely performed in the pre-TAVI work-up for appropriate prosthesis sizing and evaluation of access routes.

A previous analysis showed that obstructive CAD on CTA could be excluded in only 37% of TAVI patients if all coronary segments were evaluated [4]. Considering that only proximal coronary arteries need evaluation according to the current guidelines, we hypothesized that CTA could exclude a higher percentage of clinically relevant proximal stenosis. Therefore, we collected individual patient data from 1060 subjects from studies that investigated the diagnostic accuracy of CTA as compared to ICA to detect obstructive coronary stenosis in patients who were evaluated for TAVI. In this data set, we assessed the diagnostic yield and accuracy of pre-TAVI CTA to detect left main (LM) and proximal coronary stenosis.

## Materials and methods

### Study design, patient population, and study selection

This collaborative study contains patients who underwent both CTCA and ICA in the diagnostic work-up for TAVI. The patient population was selected through a literature search in OVID MEDLINE (including Epub Ahead of Print, In-Process & Other Non-Indexed Citations) and OVID EMBASE from January 1, 1990, to October 1, 2019. We searched for the concepts of TAVI and CTA, using controlled terms like MesH and text words. No language, date, or other restrictions were applied. The reference lists and citing articles of the identified relevant papers were cross-checked in Web of Science. Studies were considered for inclusion if they complied with the following requirements: original studies reporting on the diagnostic accuracy of CTA to detect coronary stenosis, used ICA as reference standard, reported on patients in the work-up for TAVI. Eight studies matched the criteria and the authors were approached for collaboration [5–12]. The authors of four studies could accommodate the data for a per-segment analysis [5–8]. All included patients have been previously reported [5–8]. These prior articles reported on the diagnostic accuracy of CTA, as compared to ICA, to detect obstructive CAD in all coronary segments whereas in this manuscript we report on the diagnostic accuracy of CTA, as compared to ICA, to detect obstructive CAD in the proximal coronary segments only. All patients provided written informed consent according to the policy of each participating hospital. The methodological quality was assessed using the modified Quality Assessment of Studies of Diagnostic Accuracy Included in Systematic Reviews–2 (QUADAS-2) criteria [13].

### Data collection

The data sets included patient characteristics regarding age, sex, body mass index (BMI), and the heart rate during CT scan. Information about comorbidities included the presence of diabetes mellitus, atrial fibrillation, hypercholesterolemia, peripheral arterial disease, hypertension, smoking, and a history of CAD, PCI, and coronary artery bypass grafting. Technical CT scanner information included scanner type, number of detector rows, number of slices, detector width, CT scanner rotation time, scan protocol and settings (tube voltage and tube current), contrast agent type (concentration) and volume, dose length product, and nitroglycerin use. The data sets included stenosis grading for both CTA and ICA in the left main (LM) and the proximal segments of the right coronary artery (RCA), left anterior descending (LAD) artery, and circumflex (CX), according to the American Heart Association or Society of Cardiovascular Computed Tomography classification. Both use the same definition for the proximal segments. Proximal RCA is defined as the ostium to one-half the distance to the acute margin of the heart, the LM as the ostium to the bifurcation of the LAD artery and CX, the proximal LAD artery from the end of the LM to the first diagonal, and the proximal CX as the end of LM to the origin of the obtuse marginal. For stenosis grading, all studies used a cut-off value of ≥ 50% DS to determine the presence of obstructive CAD. Three out of four studies also reported an additional cut-off value of ≥ 70% DS [6–8].

### CTA acquisition

CTA acquisition in the included studies was performed using the following CT scanners: LightSpeed VCT XTe Scanner (GE Healthcare) [7], Somatom Definition (Siemens) [5], Somatom Definition Flash (Siemens) [8], Philips iCT (Philips Healthcare) [6]. All studies used a retrospective ECG-gated low-pitch spiral protocol, with CT scanner setting of 80–140 kilovolts (kV) and 185–600 mA per rotation (Table 1). The studies reported different contrast injection protocols with a mean total volume of 109.5 ml, ranging between 50 and 170 ml. The iodine concentration in the contrast medium varied between 300 and 400 mg I/ml, with a majority of 44.8% having 370 mg I/ml [5]. This translated to a mean total iodine load of 40.8 g iodine with standard deviation of 10.4 g iodine. The mean dose length product of the TAVI CTA was 1910 ± 616.3 mGy*cm and included all CTA sequences used for TAVI planning.

**Table 1:** Baseline table and CT characteristics table

Patient demographics	
Number of patients, n	1060
Age (years, SD)	81.7 ± 6.6
Male gender, n (%)	545 (51.4)
BMI, (kg/m^2^, SD)	26.8 ± 4.9
Diabetes mellitus, n (%)	224 (21.3)
Atrial fibrillation, n (%)	159 (15.5)
Hyperlipidemia, n (%)	543 (51.8)
Hypertension, n (%)	886 (84.0)
Heart rate during CTA, n (SD)	69.3 ± 12.8
History of PCI, n (%)	300 (29.8)
History of CABG, n (%)	162 (16.1)
**CT details and settings**	
Detector rows [width], n (%)	
40 [24]	475 (44.8)
64 [38.4]	140 (13.2)
64 [40]	330 (31.1)
256 [160]	115 (10.8)
Tube voltage (%)	
80	91 (8.6)
100	394 (37.2)
120	573 (54.1)
140	1 (0.1)
Tube current (%)	
300–400	615 (58.1)
400–600	247 (23.3)
600–800	82 (7.7)
Contrast volume (SD)	109.5 ± 20.8
Contrast concentration (%)	
300	140 (13.2)
350	115 (10.8)
370	475 (44.8)
400	330 (31.1)

Baseline characteristics and CT details and settings are listed for the patients included in the analysis. Abbreviations: *BMI*, body mass index; *CTA*, computed tomography angiography; *PCI*, percutaneous coronary intervention; *CABG*, coronary artery bypass graft

### Invasive coronary angiography acquisition

In the included studies, ICA served as the reference standard and was performed by experienced readers who were blinded for the CTA results. Three out of four studies used off-line quantitative coronary angiography for stenosis assessment and evaluated segments in at least 2 orthogonal projections [6–8]. One study evaluated coronary stenosis by visual assessment [5].

### Objectives

The primary objective was to assess the diagnostic yield and accuracy of pre-TAVI CTA to detect coronary stenosis (≥ 50% DS and ≥ 70% DS) in the LM and proximal coronary segments on a per-patient and a per-segment level. The secondary objective was to perform a subgroup analysis of the individual studies and to assess the influence of atrial fibrillation and heart rate on the diagnostic accuracy of CTA.

### Statistical analysis

Data analysis was performed using the statistical software R version 3.5.1 (R Foundation for Statistical Computing). Continuous variables were presented as means with standard deviations (SD). The distribution of continuous variables was tested with the Shapiro-Wilk test. Categorical variables were presented as frequencies and percentages. The prevalence of proximal CAD was based on ICA. Diagnostic accuracy of pre-TAVI CTA, as compared to pre-TAVI ICA, was defined as the sensitivity, specificity, positive predictive value (PPV), negative predictive value (NPV), positive likelihood ration (pos-LR), and negative likelihood ratio (neg-LR). The analysis was performed on a per-patient and per-segment level. All non-diagnostic segments were labelled as if there was a coronary stenosis. Diagnostic yield was defined as the sum of the negatives (true negatives and false negatives) and presented for both the total of patients and for a subgroup without patients with CABG. For subgroup analysis, we defined subgroups of patients with and without atrial fibrillation as well as subgroups with a heart rate < 70 and ≥ 70 beats/min. The diagnostic accuracy in these subgroups was assessed on a per-patient level for stenosis ≥ 50% DS. Diagnostic accuracy measures were compared using the Pearson’s chi-squared test statistic.

## Results

### Baseline characteristics

**Fig. 1:**
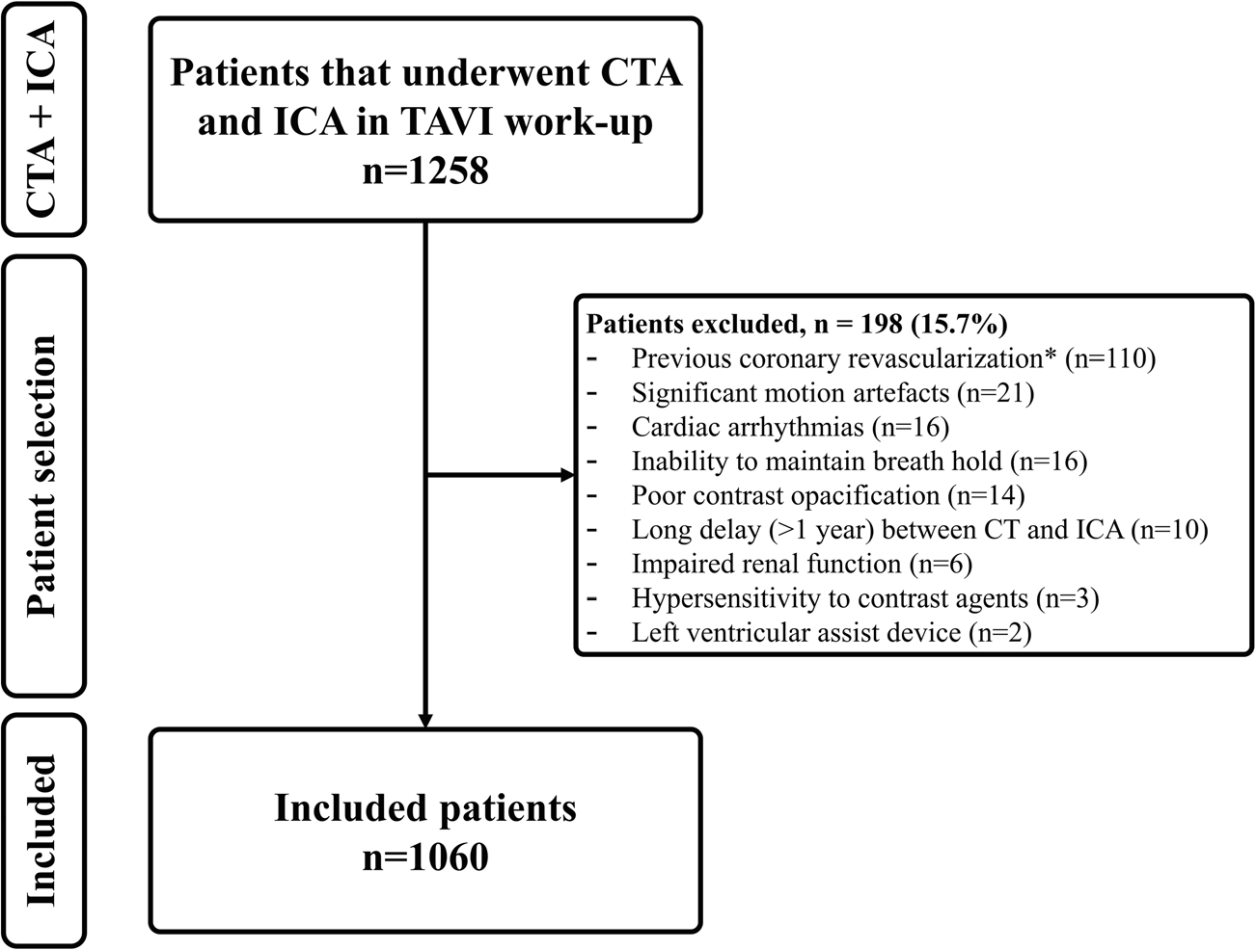
Flowchart of patient selection in the included patient population. A total of 1060 out of 1258 patients who underwent both CTA and ICA in the work-up for TAVI were included in the final analysis of the individual studies. Reasons for exclusion of patients are listed. Abbreviations: ICA, invasive coronary angiography; CTA, computed tomography angiography; TAVI, transcatheter aortic valve implantation

Patient selection is summarized in Fig. 1 and baseline and CT scan characteristics are listed in Table 1. The combined studies included 1060 patients with a mean age of 81.7 ± 6.6 years and 42.7% of patients were male. The mean BMI was 26.8 ± 4.9 kg/m^2^, diabetes mellitus was present in 28.3% of our study population, and 24.7% had atrial fibrillation. A total of 300 patients (29.8%) had prior percutaneous coronary intervention (PCI) and 16.1% had previous coronary artery bypass grafting. Mean heart rate during pre-TAVI CTA was 69.3 beats/min and varied between 61/min and 74/min in the included studies. Methodological quality assessment of included studies by QUADAS-2 is discussed in supplementary material (supplemental text and supplemental table 1 and supplemental figure 1).

### Prevalence of obstructive proximal CAD at ICA

Using ICA as a reference standard, proximal stenosis of ≥ 50% DS was present in 307 of 1060 patients (29.0%), with coronary stenosis located in the proximal RCA (n = 193, 18.2%), the LM coronary artery (n = 47, 4.4%), the proximal LAD artery (n = 162, 15.3%), and the proximal CX (n = 120, 11.3%). The prevalence differed between the populations included in the individual studies. The lowest prevalence was 15.7% in the study of Rossi et al [8], followed by 18.8% in the study of Andreini et al [5], 32.2% in the study of Hamden et al [7], and 39.2% in the study of Opolski et al [6].

In a subgroup of 585 patients, data was also available for an additional coronary stenosis threshold ≥ 70% DS. Proximal coronary stenosis ≥ 70% DS was present in 92 of 585 patients (15.7%), with coronary stenosis located in the proximal RCA (n = 57, 9.7%), the LM (n = 4, 0.7%), the proximal LAD artery (n = 46, 7.9%), and the proximal CX (n = 27, 4.6%).

### Accuracy of CTA for the detection of obstructive proximal CAD

#### Stenosis ≥ 50% DS

**Fig. 2:**
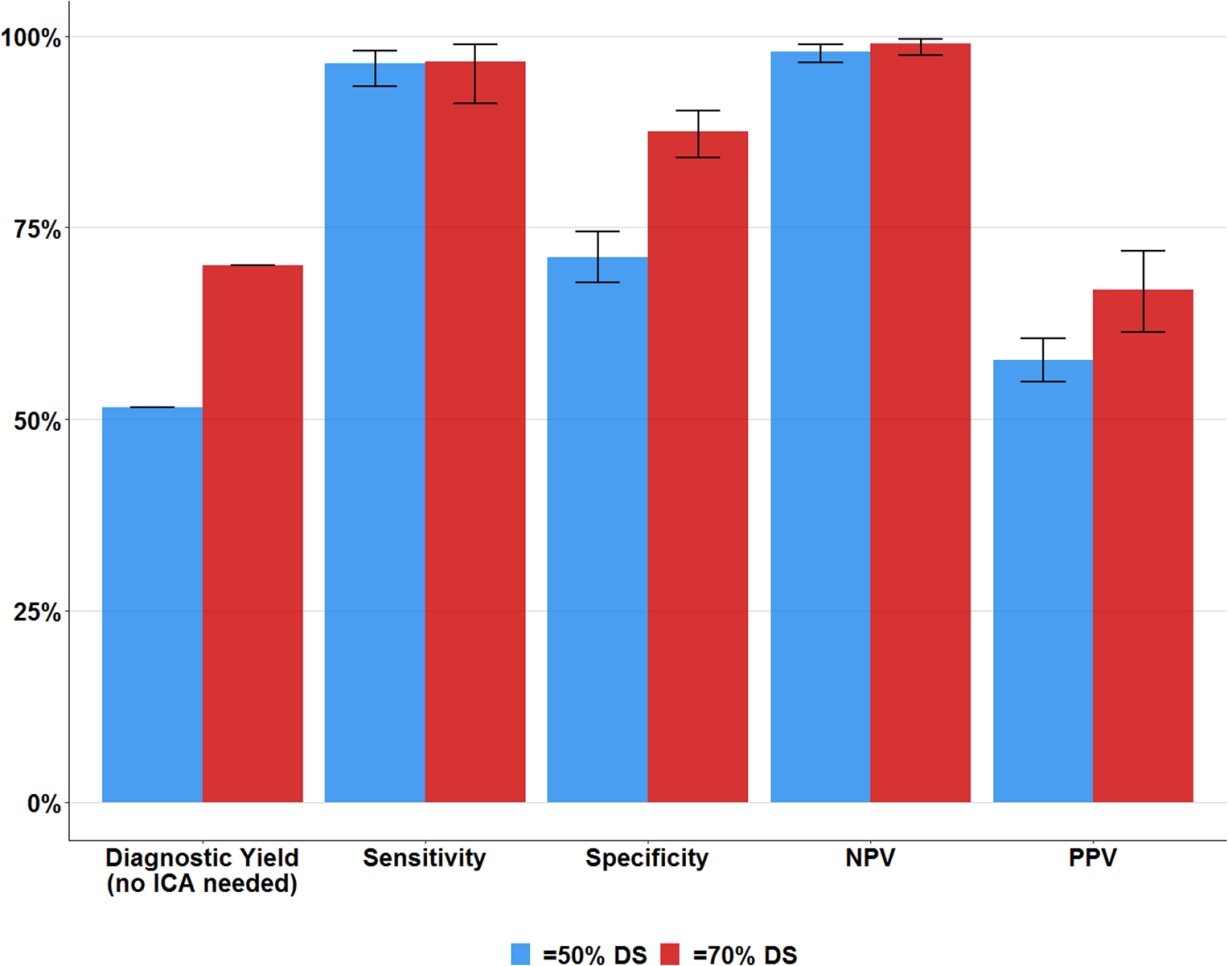
Diagnostic yield and accuracy of CTA to detect proximal coronary stenosis, using a threshold of ≥ 50% and 70% diameter stenosis. Abbreviations: ICA, invasive coronary angiography; CTA, computed tomography angiography; NPV, negative predictive value; PPV, positive predictive value; TAVI, transcatheter aortic valve implantation

The diagnostic accuracy for the detection of proximal ≥ 50% DS is listed in Table 2 and summarized in Fig. 2. The diagnostic yield of CTA by ruling out proximal ≥ 50% DS was 51.6% (547 patients, 536 true negatives + 11 false negatives). The consistency rate of CTA and ICA was 78.5%. The sensitivity and NPV of CTA to detect ≥ 50% DS were 96.4% and 98.0%, respectively. The specificity and the PPV were 71.2% and 57.7%, respectively. In the 513 (48.4%) patients in whom coronary stenosis was not ruled out, CTA showed proximal ≥ 50% DS in 315 patients (29.7%) and non-evaluable proximal segments in 198 patients (18.7%). All corresponding standard deviations and positive and negative likelihood ratios (LR) are listed in Table 2, together with the location of coronary stenosis and non-evaluable segments. In the 898 patients without CABG, the diagnostic yield was 56.6% (508, 499 true negatives + 9 false negatives). On a per-segment level (including the non-evaluable segments), CTA correctly classified 89.7% of all proximal segments. CTA correctly identified 460 out of 506 proximal coronary stenosis ≥ 50% DS, resulting in a sensitivity of 90.9%. Of the 3733 segments without obstructive stenosis, CTA correctly ruled out obstructive stenosis in 3342 segments, resulting in a specificity of 89.5%. The PPV was 54.1% and the NPV was 98.6%.

**Table 2:** Diagnostic accuracy in proximal stenosis of ≥ 50% DS

	Total	TP	TN	FP	FN	Sensitivity (%)	Specificity (%)	PPV (%)	NPV (%)	Pos-LR	Neg-LR
Per patient	1060	296	536	217	11	96.4 (93.5–98.1)	71.2 (67.8–74.4)	57.7 (54.9–60.5)	98.0 (96.5–98.9)	3.34 (2.98–3.75)	0.05 (0.03–0.09)
Per segment											
-All segments(with non-evaluable)	4239	460	3342	391	46	90.9 (88.6–93.2)	89.5 (88.5–90.5)	54.1 (50.6–57.4)	98.6 (98.2–99.0)	8.68 (7.87–9.57)	0.10 (0.08–0.13)
Evaluable segments CTA	Total	TP	TN	FP	FN	Non-evaluable segments CTA		Total	ICA positive	ICA negative	ICA ND
-Proximal RCA	971	146	736	73	16	-Proximal RCA		89	13	76	0
-Left main	1028	29	934	57	8	-Left main		31	10	21	0
-Proximal LAD	992	120	706	150	16	-Proximal LAD		68	26	41	1
-Proximal rCX	977	82	778	111	6	-Proximal rCX		83	32	50	1

Diagnostic accuracy of CTA as compared with invasive coronary angiography for the detection of > 50% DS coronary lesions. Values between parentheses represent the outer bounds of the 95% confidence interval. Abbreviations: *TP*, true positives; *TN*, true negatives; *FN*, false negatives; *FP*, false positives; *PPV*, positive predictive value; *NPV*, negative predictive value; *Pos-LR*, positive likelihood ratio; *Neg-LR*, negative likelihood ratio; *ICA*, invasive coronary angiography; *RCA*, right coronary artery; *LAD*, left anterior descending artery; *rCX*, ramus circumflex; *ND*, non-evaluable

#### Stenosis ≥ 70% DS

Data to calculate the diagnostic accuracy for the detection of proximal ≥ 70% DS was available in 585 patients (Table 3, Fig. 2). The diagnostic yield of CTA to rule out proximal ≥ 70% DS was 70.1% (410 patients, 406 true negatives + 4 false negatives). The consistency rate of CTA and ICA was 89.4%. The sensitivity and NPV of CTA to detect ≥ 70% DS were 96.7% and 99.0%, respectively. The specificity and the PPV were 87.5% and 66.9%, respectively. In the 496 patients without CABG, the diagnostic yield was 72.2% (358, 356 true negatives + 2 false negatives).

**Table 3:** Diagnostic accuracy in proximal stenosis of ≥ 70% DS

	Total	TP	TN	FP	FN	Sensitivity (%)	Specificity (%)	PPV (%)	NPV (%)	Pos-LR	Neg-LR
Per patient	585	117	406	58	4	96.7 (91.2–98.9)	87.5 (84.1–90.3)	66.9 (61.3–72.0)	99.0 (97.5–99.6)	7.74 (6.07–9.86)	0.04 (0.01–0.10)
Per segment											
-All segments(with non-evaluable)	2257	108	2107	27	15	87.8 (80.4–92.8)	98.7 (98.1–99.1)	80.0 (72.1–86.2)	99.3 (98.8–99.6)	69.4 (47.4–102)	0.12 (0.08–0.20)
Evaluable segments CTA	Total	TP	TN	FP	FN	Non-evaluable segments CTA		Total	CAG positive	CAG negative	CAG ND
-Proximal RCA	554	48	498	4	4	-Proximal RCA		31	6	25	0
-Left main	580	2	573	3	2	-Left main		5	0	5	0
-Proximal LAD	563	37	510	9	7	-Proximal LAD		22	3	19	0
-Proximal rCX	560	21	526	11	2	-Proximal rCX		25	0	21	0

Diagnostic accuracy of CTA as compared with invasive coronary angiography for the detection of > 70% DS coronary lesions. Values between parentheses represent the outer bounds of the 95% confidence interval. Abbreviations: *TP*, true positives; *TN*, true negatives; *FN*, false negatives; *FP*, false positives; *PPV*, positive predictive value; *NPV*, negative predictive value; *Pos-LR*, positive likelihood ratio; *Neg-LR*, negative likelihood ratio; *RCA*, right coronary artery; *LAD*, left anterior descending artery; *rCX*, ramus circumflex; *ND*, non-evaluable

On a per-segment level (including the non-evaluable segments), CTA correctly classified 98.1% of segments, resulting in a sensitivity of 87.8%, specificity of 98.7%, PPV of 80.0%, and NPV of 99.3%, respectively. In 42 out of 2257 evaluable segments, CTA misclassified a coronary stenosis with a threshold of ≥ 70% DS. These misclassifications were the result of overestimation of the stenosis in 27 cases (false positives) and underestimation of the stenosis in 15 cases (false negatives).

### The influence of atrial fibrillation and heart rate

In the subgroup of 159 patients with atrial fibrillation, per-patient evaluation with CTA correctly identified 79.2% of patients (vs. 78.5% of correctly identified patients in the total cohort), with a sensitivity of 100%, specificity of 72.0%, PPV of 55.4%, and NPV of 99.4%. Compared to the patients with atrial fibrillation, CTA correctly identified proximal ≥ 50% DS in 74.8% of patients without atrial fibrillation (*p* = 0.27). There was also no significant difference in the diagnostic accuracy measures of the patients without atrial fibrillation (sensitivity of 95.7%, specificity of 70.2%, PPV of 56.1%, and NPV of 97.6%).

In the subgroup of 552 patients with a heart rate < 70 b/min, per-patient evaluation with CTA correctly identified 84.6% of patients, with a sensitivity of 96.3%, a specificity of 79.6%, PPV of 66.7%, and NPV of 98.1%. In the subgroup with a heart rate of ≥ 70 b/min, CTA correctly identified significantly less patients (71.5%, *p* ≤ 0.001). There was no significant difference in the sensitivity and NPV (sensitivity of 96.5% and NPV of 97.8%). However, specificity and PPV were significantly lower than in the patients with heart rate < 70 b/min (specificity of 61.8%, *p* ≤ 0.001 and PPV of 49.6%, *p* ≤ 0.001)

## Discussion

Our findings demonstrate that assessment of proximal stenosis in the TAVI work-up by CTA can be used as a gatekeeper for ICA in routine clinical practice. The highly sensitive CTA is capable to rule out proximal ≥ 50% DS in 52% of patients and proximal ≥ 70% DS in 70% of patients, hereby substantially reducing the need for ICA in the work-up for TAVI. However, the relative low specificity and PPV may limit the clinical utility and applicability of CTA to guide coronary revascularization in the TAVI candidates.

Pre-TAVI screening for CAD is required to estimate baseline procedural risk and indicate if patients need revascularization prior to TAVI. The prevalence of obstructive CAD in all coronary segments ranges from 40 to 70% according to several large multicenter registries such as the FRANCE 2 and the PARTNER TAVI registries [2]. However, the prognostic role of CAD in patients undergoing TAVI remains unknown and the most recent guidelines recommend that revascularization should only be considered in proximal coronary stenosis ≥ 70% DS [3]. If only the proximal coronary segments are evaluated, only a minority of patients would qualify for obstructive CAD. In our cohort, proximal obstructive CAD was only prevalent in 29.0% of patients on the basis of a ≥ 50% DS cut-off value and 15.7% of patients on the basis of a ≥ 70% DS cut-off value. To our knowledge, the only other study that reported a prevalence of proximal coronary artery stenosis ≥ 50% DS in TAVI patients, found a prevalence of 17% [14]. That study excluded patients with a history of acute coronary syndrome or previous coronary revascularization, causing bias and lowering the prevalence of obstructive proximal CAD.

Between the studies that were included in this collaborative study, the prevalence of obstructive proximal CAD ranged between 15.7 and 39.2%. The study by Rossi et al excluded all patients with prior coronary revascularization (a total of 110 patients, 44% of total), resulting in the lowest prevalence of obstructive proximal CAD (15.7%). On the contrary, the study by Opolski et al reported the lowest percentage of excluded patients (6.9%) and the highest prevalence of obstructive proximal CAD (39.2%)[5]. These differences could have led to the differences in overall diagnostic accuracy. Besides prevalence, the difference in diagnostic accuracy of CTA can be explained by the differences in CT scanner technology. The studies with the least advanced CT scanners had the lowest overall diagnostic accuracy [5, 7]. Conversely, studies with the most advanced CT scanners had the highest overall diagnostic accuracy [6, 8].

We tested the influence of previous or current atrial fibrillation and heart rate during CT acquisition on the diagnostic accuracy of CTA. The diagnostic accuracy of CTA in the patients with atrial fibrillation was comparable to patients in the overall cohort and should therefore not be considered a limitation for clinical implementation. Sensitivity, NPV, and diagnostic yield for ruling out proximal > 50% DS were not statistically different between patients with a heart rate > 70 b/min versus < 70 b/min. However, a heart rate of < 70 b/min during CTA was associated with a significantly improved specificity and PPV, compared to the patients with heart rate of ≥ 70 b/min, most likely due to more motion artifacts in the latter group. Two out of four included studies applied heart rate control using negatively chronotropic medication resulting in a mean heart rate of 63.7 b/min, compared to 73.4 b/min in the other studies [6, 7]. Therefore, heart rate control in TAVI patients could potentially improve diagnostic accuracy of CTA to detect coronary stenosis. Besides heart rate control, the use of nitroglycerin could increase the diagnostic accuracy of CTCA in TAVI patients. However, the risk of decreased blood pressure in these fragile patients with aortic valve pathology is controversial and may raise the need for dedicated monitoring during CTA acquisition.

## Clinical implications

Since CTA might be used for both CAD screening and pre-procedural TAVI planning, the combined clinical use in the TAVI work-up seems practical. Our results indicate that additional ICA could be avoided in 52% and 70% of patients in a relatively frail TAVI population if thresholds of ≥ 50% DS and ≥ 70% DS were used, respectively. TAVI patients are generally at increased age and have increased risk of bleeding and of vascular, embolic, and neurological complications [15, 16]. Major vascular complications were registered in 3.6% of elderly patients undergoing diagnostic ICA [17]. Therefore, avoiding ICA could reduce complications in the TAVI work-up. Reducing the number of diagnostic tests that use nephrotoxic contrast material is desirable in a patient population that is at increased risk of contrast-induced acute kidney injury [18].

In clinical practice, two cut-off values for obstructive coronary stenosis are widely used (≥ 50% DS and ≥ 70% DS). Currently, there are no studies available investigating the effect of PCI in individual patients with proximal obstructive stenosis (≥ 70% DS), or patients with functional or hemodynamic significant stenosis. The effect of pre-TAVI PCI in this stenosis has to be elucidated. The preliminary results of the randomized ACTIVATION trial suggest that pre-TAVI revascularization is not linked to changes in 1 year outcomes after TAVI [19]. Until these final results become available, we recommend to maintain a 50% DS cut-off value as a safety margin and to discuss all patients with proximal coronary stenosis of ≥ 50% DS in a multidisciplinary heart valve team to determine indications for additional ICA.

## Limitations

This study is an analysis of patient data, generated from four single-center observational studies, which mainly included unselected TAVI patients. One study excluded patients with a history of prior coronary revascularization, potentially influencing the prevalence of CAD. The other studies used inclusion and exclusion criteria that may have resulted in the selection of patients with better image quality CT scans and thus overestimate the diagnostic accuracy (exclusion of patients with cardiac arrhythmias, left ventricular assist device, significant motion artifacts on CTA, or poor contrast opacification). Another limitation of this study is that all CTA scans in the individual studies were acquired with older generation CT scanners. Modern CT scanners would likely have increased the diagnostic yield and accuracy to detect proximal CAD in TAVI patients. Furthermore, all included studies used different contrast injection protocols and different contrast media concentrations. Due to the small number of studies with differences in both CT scanner settings and contrast injection parameters, it is impossible to formulate recommendations on contrast injection protocols or specific contrast media to improve diagnostic accuracy.

## Conclusion

CTA provides high diagnostic accuracy to rule out LM and proximal coronary stenosis in patients undergoing work-up for TAVI. Clinical application of CTA as a gatekeeper for ICA would reduce the need for ICA in 52% or 70% of patients, using a threshold of ≥ 50% or ≥ 70% DS, respectively.

## Supplementary information

ESM 1 Supplemental figure 1. Summary of QUADAS II. Risk of bias and applicability concerns are represented as low risk (green) or high risk (red). QUADAS-II = Quality Assessment of Studies of Diagnostic Accuracy Included in Systematic Reviews 2 (DOCX 142 kb)

## Abbreviations

BMI: Body mass index
CABG: Coronary artery bypass grafting
CAD: Coronary artery disease
CT: Computed tomography
CTA: Computed tomography angiography
CX: Circumflex
DS: Diameter stenosis
ICA: Coronary angiography
LAD: Left anterior descending
LM: Left main
MDCT: Multidetector computed tomography
neg-LR: Negative likelihood ratio
NPV: Negative predictive value
PCI: Percutaneous coronary intervention
pos-LR: Positive likelihood ration
PPV: Positive predictive value
QUADAS-2: Quality Assessment of Studies of Diagnostic Accuracy Included in Systematic Reviews–2
RCA: Right coronary artery
SD: Standard deviation
TAVI: Transcatheter aortic valve implantation

## Declarations

### Guarantor

The scientific guarantor of this publication is Nils Planken.

### Statistics and biometry

One of the authors has significant statistical expertise.

### Study subjects or cohorts overlap

Some study subjects or cohorts have been previously reported in previous studies, as described in the manuscript. The previous studies described the diagnostic accuracy of CTA, as compared to invasive coronary angiography, to diagnose coronary lesions in all segments of the coronary tree in patients who underwent TAVI. The current study combines the cohorts of the previous studies and investigates the diagnostic accuracy of CTA, as compared to invasive coronary angiography, in the proximal coronary segments only.

The studies that described study subjects, included in the current cohort:

1. Andreini D, Pontone G, Mushtaq S, Bartorelli AL, Ballerini G, Bertella E, et al Diagnostic accuracy of multidetector computed tomography coronary angiography in 325 consecutive patients referred for transcatheter aortic valve replacement. *Am Heart J* [Internet]. 2014;168(3):332–9. Available from: 10.1016/j.ahj.2014.04.022

2. Rossi A, De Cecco CN, Kennon SRO, Zou L, Meinel FG, Toscano W, et al CT angiography to evaluate coronary artery disease and revascularization requirement before trans-catheter aortic valve replacement. *J Cardiovasc Comput Tomogr*. 2017;11(5):338–46.

3. Hamdan A, Wellnhofer E, Konen E, Kelle S, Goitein O, Andrada B, et al Coronary CT angiography for the detection of coronary artery stenosis in patients referred for transcatheter aortic valve replacement. *J Cardiovasc Comput Tomogr* [Internet]. 2015;9(1):31–41. Available from: 10.1016/j.jcct.2014.11.008

4. Opolski MP, Christoph WK, Linden A Van, Walther T, Hamm CW, Mo H. Diagnostic accuracy of computed tomography angiography for the detection of coronary artery disease in patients referred for transcatheter aortic valve implantation. 2015;471–80.

### Methodology

• Retrospective

• Diagnostic or prognostic

• Multicenter study
