# Supplementary material for: DEtection of ProxImal Coronary stenosis in the work-up for Transcatheter aortic valve implantation using CTA (from the DEPICT CTA collaboration)
Source: Eur Radiol. 2021 Jun 16;32(1):143–51. doi: 10.1007/s00330-021-08095-2 (PMC8660749; doi:10.1007/s00330-021-08095-2)
Supplement: Supplementary file 1 — Supplemental figure 1. Summary of QUADAS II. Risk of bias and applicability concerns are represented as low risk (green) or high risk (red). QUADAS-II = Quality Assessment of Studies of Diagnostic Accuracy Included in Systematic Reviews 2 (DOCX 142 kb) [file 330_2021_8095_MOESM1_ESM.docx]

**Supplementary Materials**

**Risk of bias within studies**

Methodological quality assessment of included studies by QUADAS-2 is summarized in figure 1 and listed for the individual studies in supplemental table 1. Risk of selection bias scored high, because the inclusion and exclusion criteria in the individual studies could have resulted in a selected patient population (1–4). Patient selection is visualized for all studies combined in a flowchart (figure 2) and listed for all studies separately (supplemental table 2). The total number of patients who underwent both pre-TAVI CTA and pre-TAVI ICA in the individual studies was 1258, but only 1060 patients were included in the final analysis (figure 1). One study excluded patients with prior known CAD (110 patients), which raised concerns regarding applicability in a real-world TAVI population (2). The other exclusion criteria were mainly focused on acquiring appropriate image quality on CTA (i.e. exclusion of CTA with motion artefacts, exclusion of patients with arrhythmias, poor contrast opacification on CTA) (88 patients). These criteria do not raise concerns about applicability of the results, but may have altered the diagnostic accuracy of the individual studies, and consequently have influenced the presented results of the analysis. All studies defined CTA as the index test and blinded CTA reviewers for ICA outcome, resulting in a low risk of interpretation bias. One study maintained a maximum interval of 1 year between CTA and ICA, with the risk of significant disease progression (3).

**References**

1. Andreini D, Pontone G, Mushtaq S, Bartorelli AL, Ballerini G, Bertella E, et al. Diagnostic accuracy of multidetector computed tomography coronary angiography in 325 consecutive patients referred for transcatheter aortic valve replacement. Am Heart J [Internet]. 2014;168(3):332–9. Available from: http://dx.doi.org/10.1016/j.ahj.2014.04.022

2. Rossi A, De Cecco CN, Kennon SRO, Zou L, Meinel FG, Toscano W, et al. CT angiography to evaluate coronary artery disease and revascularization requirement before trans-catheter aortic valve replacement. J Cardiovasc Comput Tomogr. 2017;11(5):338–46.

3. Hamdan A, Wellnhofer E, Konen E, Kelle S, Goitein O, Andrada B, et al. Coronary CT angiography for the detection of coronary artery stenosis in patients referred fortranscatheter aortic valve replacement. J Cardiovasc Comput Tomogr [Internet]. 2015;9(1):31–41. Available from: http://dx.doi.org/10.1016/j.jcct.2014.11.008

4. Opolski MP, Christoph WK, Linden A Van, Walther T, Hamm CW, Mo H. Diagnostic accuracy of computed tomography angiography for the detection of coronary artery disease in patients referred for transcatheter aortic valve implantation. 2015;471–80.

**Supplementary Figure 1**


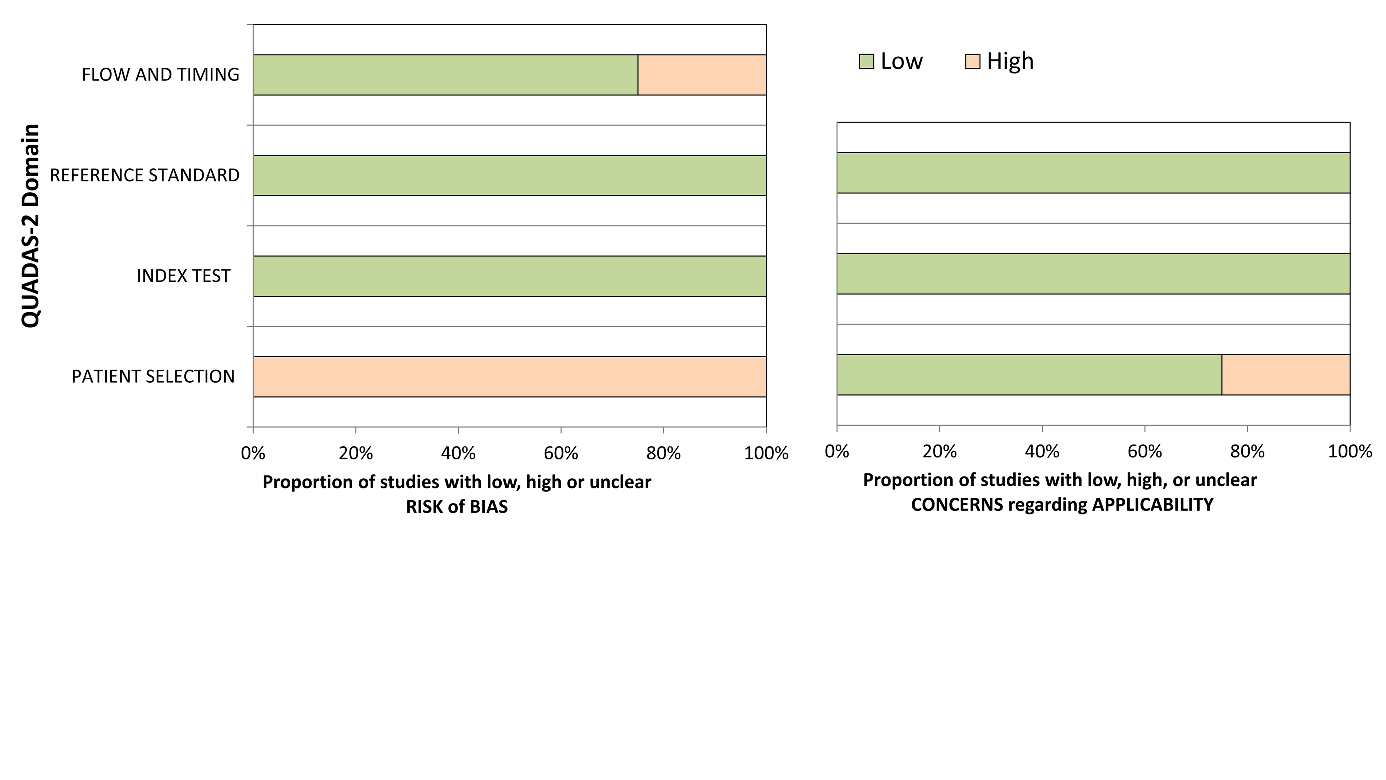


# Supplemental table 1. Methodological quality assessment of included studies by QUADAS II

|  | **Risk of Bias** | | | | **Applicability concerns** | | |
| --- | --- | --- | --- | --- | --- | --- | --- |
|  | **Patient-selection** | **Index Test** | **Reference standard** | **Flow and timing** | **Patient-selection** | **Index Test** | **Reference standard** |
| Andreini, et al. | ☹ | ☺ | ☺ | ☺ | ☺ | ☺ | ☺ |
| Hamdan, et al. | ☹ | ☺ | ☺ | ☹ | ☺ | ☺ | ☺ |
| Opolski, et al. | ☹ | ☺ | ☺ | ☺ | ☺ | ☺ | ☺ |
| Rossi, et al. | ☹ | ☺ | ☺ | ☹ | ☹ | ☺ | ☺ |

**Symbols represents the reported risk of bias or the concerns about applicability**. ☺ = low risk, ☹ = high risk. QUADAS-2 = Quality Assessment of Studies of Diagnostic Accuracy Included in Systematic Reviews 2
